# Supplementary material for: Unusual mammalian usage of TGA stop codons reveals that sequence conservation need not imply purifying selection
Source: PLoS Biol. 2022 May 12;20(5):e3001588. doi: 10.1371/journal.pbio.3001588 (PMC9129041; doi:10.1371/journal.pbio.3001588)
Supplement: S1 Fig — Autosomal size (bp length) is negatively associated with G+C content (Spearman’s rank; p = 0.0078, rho = ‒0.56, n = 22) and TGA usage (Spearman’s rank; p = 0.0094, rho = ‒0.55, n = 22). Underlying data can be found in S9 Data. (PDF) [file pbio.3001588.s001.pdf]

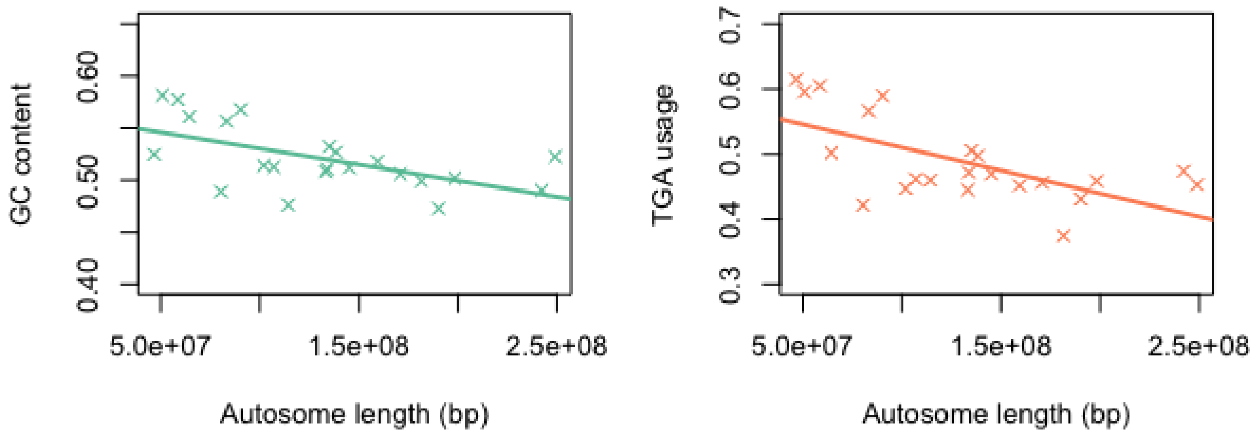

S1 Fig. The relationships of autosome length with GC content and TGA usage in the human genome. Autosomal size (bp length) is negatively associated with G+C content (Spearman's rank; $p = 0.0078$, rho = -0.56, $n = 22$) and TGA usage (Spearman's rank; $p = 0.0094$, rho = -0.55, $n = 22$). Underlying data can be found in S9 data.
