# Supplementary material for: Unusual mammalian usage of TGA stop codons reveals that sequence conservation need not imply purifying selection
Source: PLoS Biol. 2022 May 12;20(5):e3001588. doi: 10.1371/journal.pbio.3001588 (PMC9129041; doi:10.1371/journal.pbio.3001588)
Supplement: S2 Fig — The GC range used is the bottom 20% of genes to avoid the possible confounding effects of biased gene conversion. CDS refers to coding sequence, CREs to cis-regulatory elements. “xDinuc matrix” refers to equilibrium estimates of trinucleotide frequencies derived from a dinucleotide mutational matrix. Underlying data can be found in S6 Data. CDS, coding sequence; CRE, cis-regulatory element; ncRNA, noncoding RNA. (PDF) [file pbio.3001588.s002.pdf]

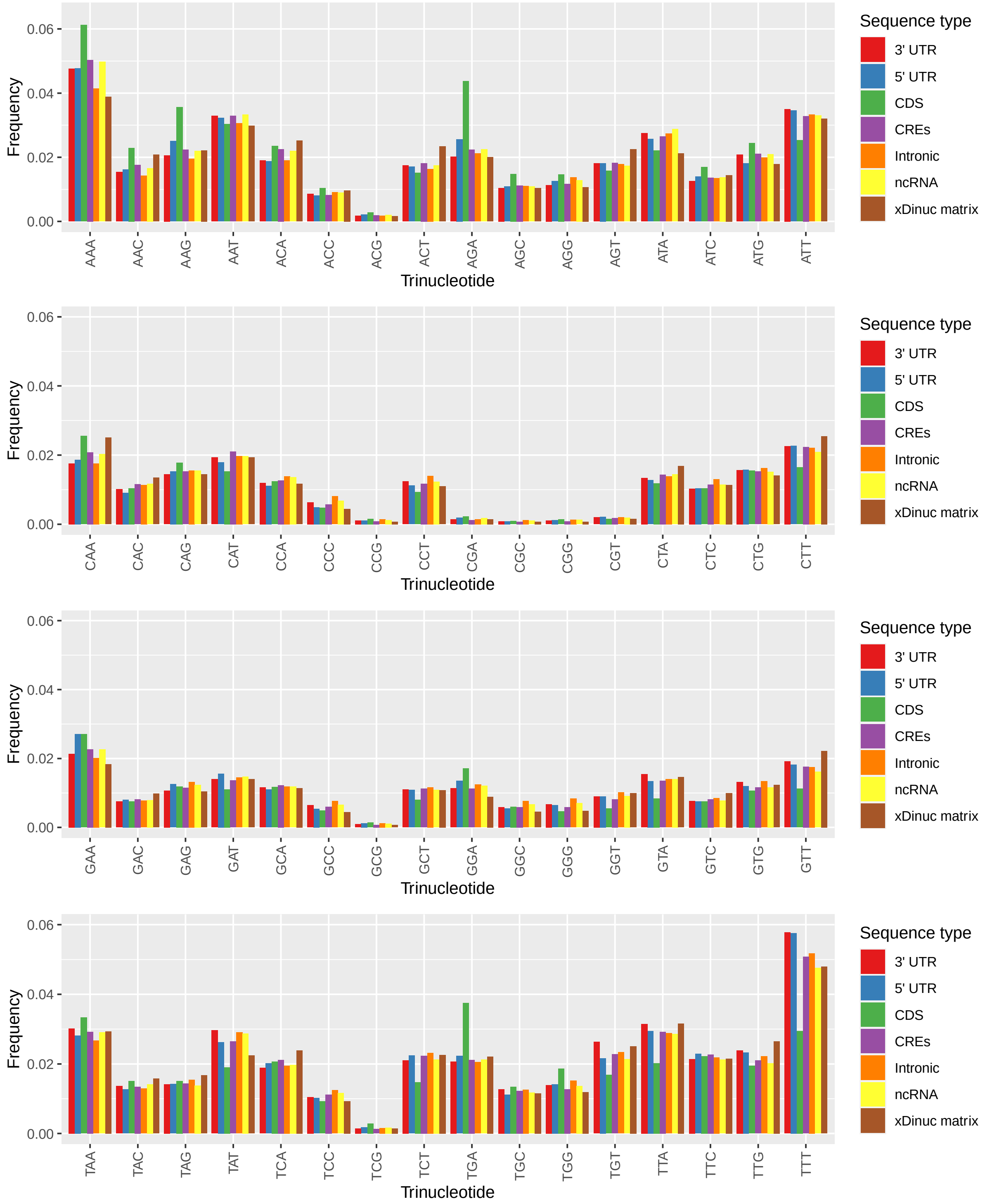

S2 Fig. Trinucleotide frequencies in six sets of different genomic sequences (between 0%-36.31% GC content) compared to dinucleotide matrix-derived equilibrium predictions. The GC range used is the bottom 20% of genes to avoid the possible confounding effects of biased gene conversion. CDS refers to coding sequence, CREs to cis-regulatory elements. “xDinuc matrix” refers to equilibrium estimates of trinucleotide frequencies derived from a dinucleotide mutational matrix. Underlying data can be found in S6 data.
