# Supplementary material for: Unusual mammalian usage of TGA stop codons reveals that sequence conservation need not imply purifying selection
Source: PLoS Biol. 2022 May 12;20(5):e3001588. doi: 10.1371/journal.pbio.3001588 (PMC9129041; doi:10.1371/journal.pbio.3001588)
Supplement: S3 Fig — Flux to the G+C-rich codons is most strongly favoured at high recombination rates (Spearman’s rank; p < 2.2 × 10‒16, rho = 0.99), consistent with the possible action of GC-biased gene conversion. Underlying data can be found in S10 Data. (PDF) [file pbio.3001588.s003.pdf]

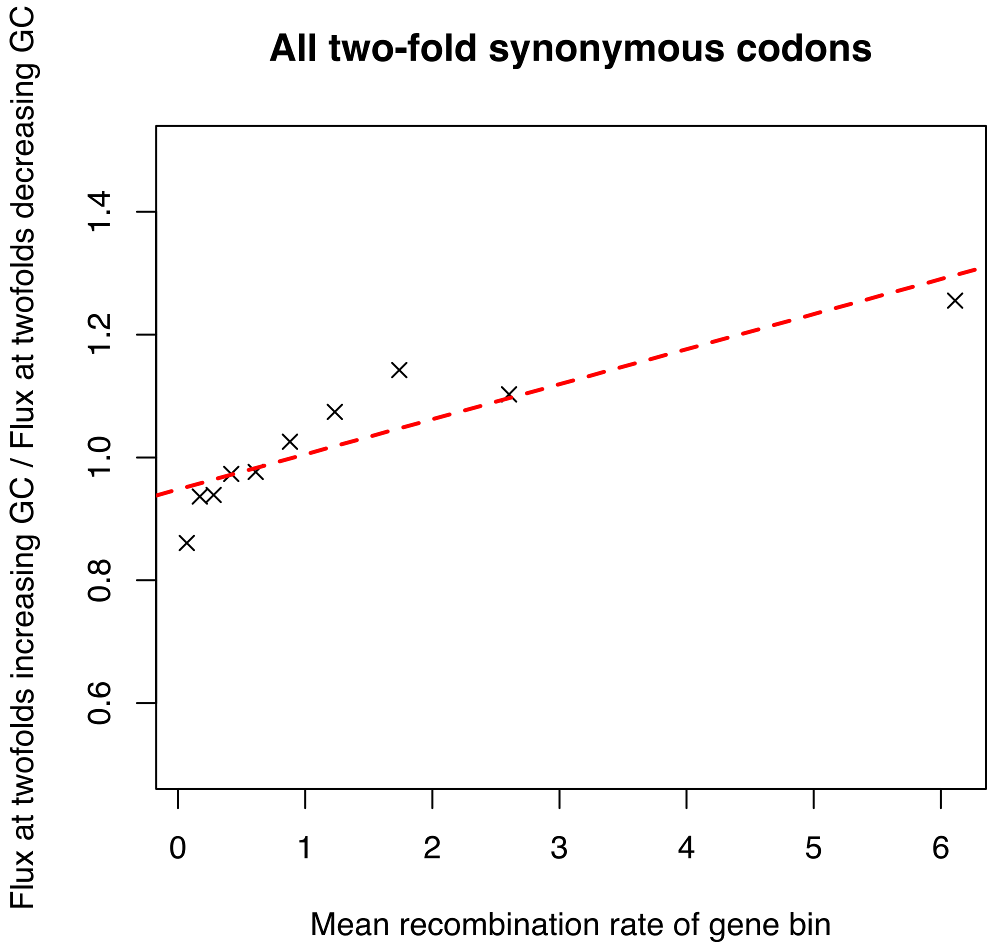

S3 Fig. The rate of flux increasing GC content at twofold degenerate sites divided by the rate of flux decreasing GC content at the same sites across 10 gene bins of increasing recombination rate. Flux to the G+C-rich codons is most strongly favoured at high recombination rates (Spearman's rank; $p < 2.2 \times 10^{-16}$, rho = 0.99), consistent with the possible action of GC-biased gene conversion. Underlying data can be found in S10 data.
