# Supplementary material for: Unusual mammalian usage of TGA stop codons reveals that sequence conservation need not imply purifying selection
Source: PLoS Biol. 2022 May 12;20(5):e3001588. doi: 10.1371/journal.pbio.3001588 (PMC9129041; doi:10.1371/journal.pbio.3001588)
Supplement: S4 Fig — Flux increasing GC content are significantly favoured in regions with higher recombination rate in 10 of the 12 amino acids before Bonferroni correction (Spearman’s rank tests; p < 0.05), the 2 exceptions to this being Leucine and Glutamine. Underlying data can be found in S10 Data. (PDF) [file pbio.3001588.s004.pdf]

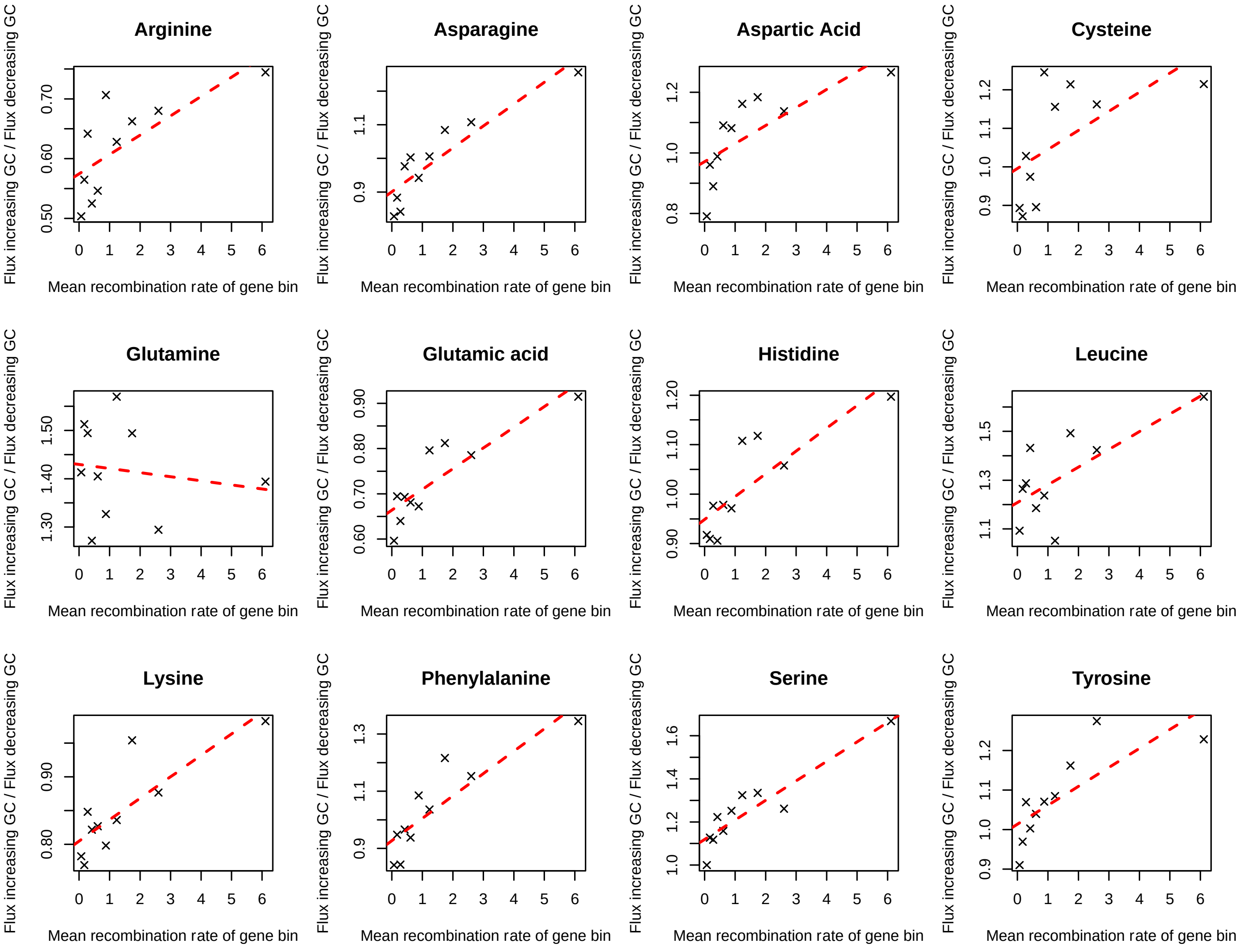

S4 Fig. The rate of flux increasing GC content at twofold degenerate sites divided by the rate of flux decreasing GC content at the same sites across 10 gene bins of increasing recombination rate for each appropriate amino acid. Flux increasing GC content are significantly favoured in regions with higher recombination rate in 10 of the 12 amino acids before Bonferroni correction (Spearman's rank tests; $p < 0.05$), the two exceptions to this being Leucine and Glutamine. Underlying data can be found in S10 data.
