# Supplementary material for: Unusual mammalian usage of TGA stop codons reveals that sequence conservation need not imply purifying selection
Source: PLoS Biol. 2022 May 12;20(5):e3001588. doi: 10.1371/journal.pbio.3001588 (PMC9129041; doi:10.1371/journal.pbio.3001588)
Supplement: S5 Fig — The normalised differences, (O-E)/E, between estimated trinucleotide mutational equilibrium frequencies (calculated from DNMs) and fixed trinucleotide frequencies (from 10 kb sequences surrounding those mutations) were calculated for GC-rich (top 20%, 45.5%–100%, “group 5”) and GC-poor (bottom 20%, 0%–36.3%, “group 1”) sequences surrounding 108,778 DNMs. As we predict, GC-rich sequences to be subjected to stronger biased gene conversion, we predict a larger differential between fixed and equilibrium frequency, D, for GC-rich trinucleotides in GC-rich sequences. The extent to which a trinucleotide is “boosted” by biased gene conversion can hence be accessed by measuring the difference, (O-E)/E, in D between the GC-richest and GC-poorest sequences. Trinucleotides are ordered from low to high according to the extent they are “boosted” by biased gene conversion. Underlying data can be found in S7 data. DNM, de novo mutation; (O-E)/E, (Observed-Expected)/Expected. (PDF) [file pbio.3001588.s005.pdf]

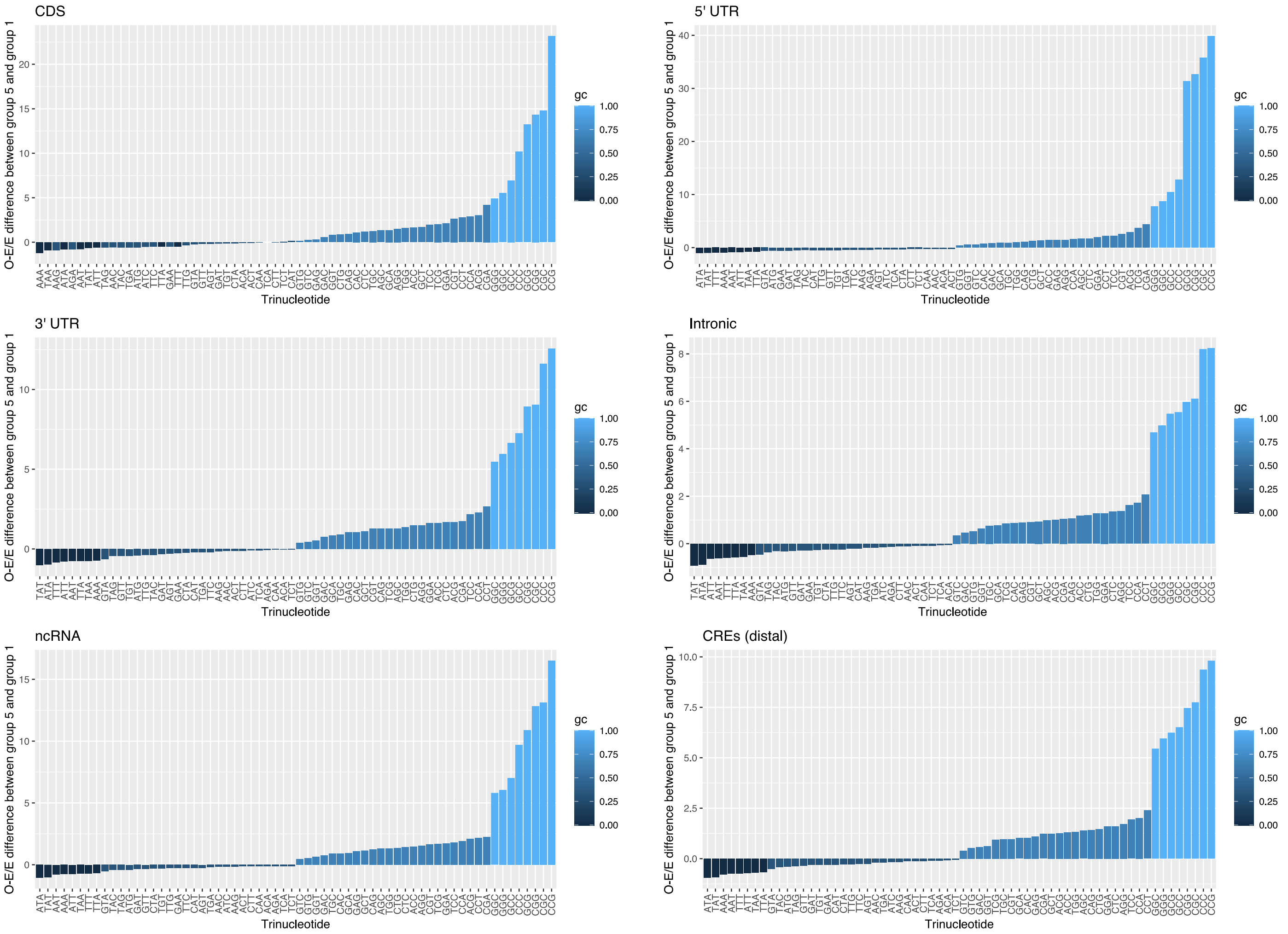

S5 Fig. Deviation scores, (O-E)/E, describing the difference in gBGC "boost" for each trinucleotide individually. The normalised differences, (O-E)/E, between estimated trinucleotide mutational equilibrium frequencies (calculated from de novo mutations, DNMs) and fixed trinucleotide frequencies (from 10kb sequences surrounding those mutations) were calculated for GC-rich (top 20%, 45.5-100%, "group 5") and GC-poor (bottom 20%, 0-36.3%, "group 1") sequences surrounding 108,778 DNMs. As we predict GC-rich sequences to be subjected to stronger biased gene conversion, we predict a larger differential between fixed and equilibrium frequency, *D*, for GC-rich trinucleotides in GC-rich sequences. The extent to which a trinucleotide is "boosted" by biased gene conversion can hence be accessed by measuring the difference, (O-E)/E, in *D* between the GC-richest and GC-poorest sequences. Trinucleotides are ordered from low to high according to the extent they are "boosted" by biased gene conversion. Underlying data can be found in S7 data.
