# Supplementary material for: Unusual mammalian usage of TGA stop codons reveals that sequence conservation need not imply purifying selection
Source: PLoS Biol. 2022 May 12;20(5):e3001588. doi: 10.1371/journal.pbio.3001588 (PMC9129041; doi:10.1371/journal.pbio.3001588)
Supplement: S6 Fig — TGA receives a consistent higher GC-coupled fixation boost than TAG which performs the second worst (after GTA). TCA similarly receives a consistently higher GC-coupled fixation boost than TAC. Sequences analysed include CRE, 5′ UTR (5), intronic (intron), ncRNA, (ncrna), and 3′ UTR (3). CDS sequences are excluded from this analysis as they are much more prone to selection and other potential fixation biases. Underlying data can be found in S8 data. CDS, coding sequence; CRE, cis-regulatory element; gBGC, GC-biased gene conversion; ncRNA, noncoding RNA. (PDF) [file pbio.3001588.s006.pdf]

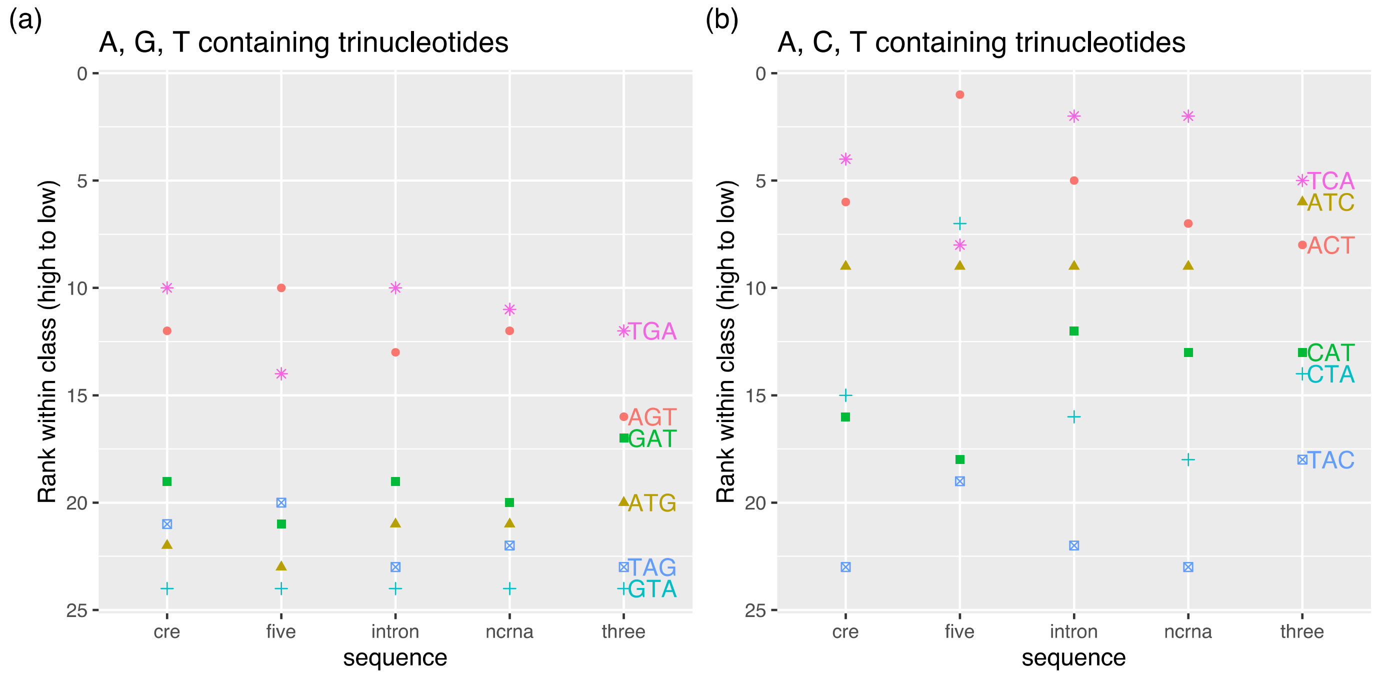

S6 Fig. Trinucleotides containing (a) A, G and T and (b) A, C and T within the 33% GC-content class of trinucleotides ranked by GC-biased gene conversion (gBGC) "boost" scores. TGA receives a consistent higher GC-coupled fixation boost than TAG which performs the second worst (after GTA). TCA similarly receives a consistently higher GC-coupled fixation boost than TAC. Sequences analysed include cis-regulatory elements (cre), 5' UTR (five), intronic (intron), ncRNA, (ncrna), and 3' UTR (three). CDS sequences are excluded from this analysis as they are much more prone to selection and other potential fixation biases. Underlying data can be found in S8 data.
