# Supplementary material for: Unusual mammalian usage of TGA stop codons reveals that sequence conservation need not imply purifying selection
Source: PLoS Biol. 2022 May 12;20(5):e3001588. doi: 10.1371/journal.pbio.3001588 (PMC9129041; doi:10.1371/journal.pbio.3001588)
Supplement: S7 Fig — Mutations were assigned a recombination rate based upon their local 10 kbp environment. Mutations in nonrecombining regions were discarded. The remaining mutations were split into bins of equal size (approximately 5,000 mutations) for the calculation of GC* and TGA*. Recombination rate is not correlated with GC* (Spearman’s rank; p = 0.58, rho = ‒0.2) nor TGA* (Spearman’s rank; p = 0.63, rho = ‒0.18) when estimated from de novo mutations. Underlying data can be found in S11 Data. (PDF) [file pbio.3001588.s007.pdf]

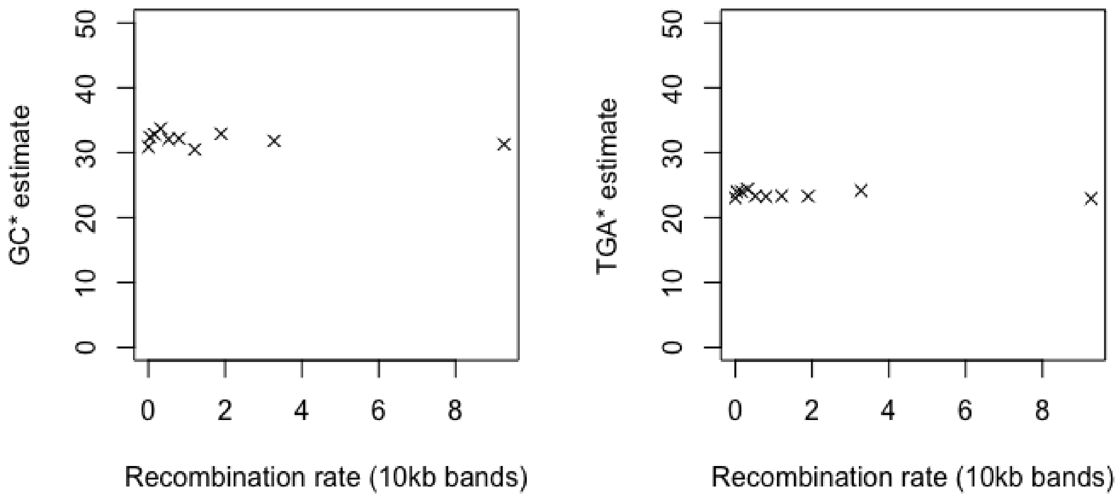

S7 Fig. Predicted G+C equilibrium (G+C*) and TGA equilibrium (TGA*) frequencies from *de novo* mutations of various recombination rates. Mutations were assigned a recombination rate based upon their local 10kbp environment. Mutations in non-recombining regions were discarded. The remaining mutations were split into bins of equal size (~5,000 mutations) for the calculation of GC* and TGA*. Recombination rate is not correlated with GC* (Spearman's rank; p = 0.58, rho = -0.2) nor TGA* (Spearman's rank; p = 0.63, rho = -0.18) when estimated from *de novo* mutations. Underlying data can be found in S11 data.
