# Supplementary material for: Unusual mammalian usage of TGA stop codons reveals that sequence conservation need not imply purifying selection
Source: PLoS Biol. 2022 May 12;20(5):e3001588. doi: 10.1371/journal.pbio.3001588 (PMC9129041; doi:10.1371/journal.pbio.3001588)
Supplement: S1 Table — (PDF) [file pbio.3001588.s008.pdf]

S1 Table. Results of linear regression models predicting stop codon (TAA, TGA, TAG) trinucleotide usage as a function of intronic G+C content in 5' and 3' UTR sequences and as a function of coding sequence GC3 content in intronic sequences.

**Sequence**	**Model**	**Estimate**	**P-value**
5' UTR	TAA ~ Intronic G+C	-0.524365	<2e-16
	TGA ~ Intronic G+C	0.544688	<2e-16
	TAG ~ Intronic G+C	-0.02032	0.116
3' UTR	TAA ~ Intronic G+C	-0.688116	<2e-16
	TGA ~ Intronic G+C	0.715928	<2e-16
	TAG ~ Intronic G+C	-0.027812	3.52e-06
Intronic	TAA ~ Coding sequence GC3	-0.015645	<2e-16
	TGA ~ Coding sequence GC3	0.01354	<2e-16
	TAG ~ Coding sequence GC3	0.0007857	0.598
