# Supplementary material for: Unusual mammalian usage of TGA stop codons reveals that sequence conservation need not imply purifying selection
Source: PLoS Biol. 2022 May 12;20(5):e3001588. doi: 10.1371/journal.pbio.3001588 (PMC9129041; doi:10.1371/journal.pbio.3001588)
Supplement: S2 Table — Rates are defined as the number of observed changes per incidence of the nucleotide in the reference genome. Under the assumption that the observed number of mutations is a Poisson variable, 95% confidence intervals (CI) were calculated using the Poisson.test function in R. (PDF) [file pbio.3001588.s009.pdf]

S2 Table. The 4 x 4 mutational matrix for 108,778 observed de novo mutations in 1,548 human trios. Rates are defined as the number of observed changes per incidence of the nucleotide in the reference genome. 95% confidence intervals (C.I.) were calculated using the Poisson.test function in R under the assumption that the observed number of mutations is a Poisson variable.

Reference allele	Estimate	Derived allele			
		A	T	C	G
A	Rate	-	3.87×10^{-6}	4.14×10^{-6}	1.56×10^{-5}
	Upper C.I.	-	4.00×10^{-6}	4.28×10^{-6}	1.58×10^{-5}
	Lower C.I.	-	3.74×10^{-6}	4.01×10^{-6}	1.53×10^{-5}
T	Rate	4.05×10^{-6}	-	1.56×10^{-5}	4.06×10^{-6}
	Upper C.I.	4.18×10^{-6}	-	1.59×10^{-5}	4.19×10^{-6}
	Lower C.I.	3.91×10^{-6}	-	1.54×10^{-5}	3.93×10^{-6}
C	Rate	6.47×10^{-6}	3.55×10^{-5}	-	8.08×10^{-6}
	Upper C.I.	6.67×10^{-6}	3.60×10^{-5}	-	8.31×10^{-6}
	Lower C.I.	6.27×10^{-6}	3.51×10^{-5}	-	7.85×10^{-6}
G	Rate	3.55×10^{-5}	6.32×10^{-6}	8.09×10^{-6}	-
	Upper C.I.	3.59×10^{-5}	6.53×10^{-6}	8.32×10^{-6}	-
	Lower C.I.	3.50×10^{-5}	6.12×10^{-6}	7.87×10^{-6}	-
