# Supplementary material for: Unusual mammalian usage of TGA stop codons reveals that sequence conservation need not imply purifying selection
Source: PLoS Biol. 2022 May 12;20(5):e3001588. doi: 10.1371/journal.pbio.3001588 (PMC9129041; doi:10.1371/journal.pbio.3001588)
Supplement: S1 Text — (PDF) [file pbio.3001588.s010.pdf]

S1 Text. Possible selective explanations for TAG avoidance compared with TGA.

TAG receives less of a “boost” in GC rich domains that TGA. Why is this? As the metric starts by specifying the expected trinucleotide abundance given known mutational profiles, we can eliminate mutation bias, unless it is even more complex than we permitted. Instead, the data support complex fixation biases. Fixation bias may imply selection for or against certain *k*-mers. Why might TAG, and its dinucleotides TA and AG, be selectively avoided in the genome? A parsimonious rationale should explain why TA dinucleotides appear to be under-represented near-universally (1) and why, as we observe, the effect is seen in transcribed and (what we presume to be) untranscribed domains (cis-regulatory elements).

Such generality might point to DNA’s biophysics, for example assumption of A and B forms. However, within the 33% trinucleotide class the two least boosted trinucleotides, TAG and GTA predispose to B and A form respectively (2). Similarly, TAC and TAG predispose to opposite forms but have comparable low fixation bias and, indeed, when we correlate A-DNA propensity energy (APE) against our gBGC “boost” scores for each trinucleotide, we find no significant correlations in 5’ UTR, 3’ UTR, intronic, ncRNA, or cis-regulatory element sequences (Spearman’s rank tests, all $p > 0.05$).

An alternative DNA structural hypothesis is that TA (or certain TA-containing oligonucleotides) might adversely affect chromatin structure (1), probably because AT-rich DNA tends to be concentrated in the nucleosome-free regions associated with transcription start sites. However, bacteria don’t have nucleosomes but nonetheless avoid TAG (3, 4). TA might also be avoided due to selection against UpA motifs on RNA molecules that are targeted by ribonucleases such as RNase L during the antiviral immune response (5, 6). However, non-transcribed domains show the same tends and why bacteria also avoid TAG is unexplained. A similar problem faces the notion of “AG exclusion zones” that are important for splicing accuracy (7, 8). Whether this could explain genome-wide avoidance of AG dinucleotides seems unlikely given trends in non-transcribed domains. Splicing is also of little relevance in bacteria.

Perhaps the most compelling model is one proposing avoidance of transcription initiation motifs. TA dinucleotides could lead to accidental incidences of, for example, “TATA” boxes in eukaryotes and “Pribnow” boxes in bacteria (i.e. the TATAA motif). More generally, TA features in many key regulatory motifs that would be inappropriate in most DNA regions in both eukaryotic and prokaryotic genomes (9, 10). Indeed, within the trinucleotides with only A and T, ATA and TAT, the two that are core to TATA box, are consistently the two with the lowest “boost” (Supplementary fig 6). However, a TATA box is classically TATA[A|T]A[A|T]. Why such a motif would select against TAG but not TAA (in the 0% GC class TAA typically has a high boost) is not clear. We need also to be weary of post hoc hypothesising. Indeed, one might also predict selection against CAA or AAT owing to their involvement in CAAT boxes, commonly located about 150 bp 5’ of TATA boxes. We see no evidence for either, CAA indeed being one of the most “boosted” of the 33% GC class and AAT being unexceptional.

Perhaps the most important objection to any such model is that one must suppose efficient selection against a point mutation causing spurious transcription or sequestration of TATA-binding protein which, when population sizes are small (e.g mammals and birds), seems unlikely. In bacteria and archaea, the strength of selection against such spurious binding is estimated to be around $N_e s = -0.09$ and thus within the range of nearly neutral mutations

for these species (11). If then *Escherichia coli*'s N_e is of the order of 10^8 (12), then *s* must be ~$-0.09/10^8 = -9 \times 10^{-10}$. For a mutation to be under selection *s* ~ 1/2 N_e must hold. In a species with N_e ~ 10,000 (e.g. humans) then this value of *s* (i.e. 1/20,000) is much greater than 9×10^{-10} estimated for selection against spurious binding. Thus, unless the selective cost of spurious binding is very much greater in humans than in bacteria, it is hard to see how selection can be efficient enough to remove spurious binding sites.

More generally, in principle the fixation bias associated with high recombination rates could be compatible with some form of nucleotide level selection that prefers G+C residues. However, *a priori* such selective models are hard to reconcile with inefficient selection associated with low N_e, so in turn non-selective fixation biases appear more parsimonious.

References

1. Burge C, Campbell AM, Karlin S. Over-representation and under-representation of short oligonucleotides in DNA sequences. Proc Natl Acad Sci USA. 1992;89(4):1358-62.
2. Basham B, Schroth GP, Ho PS. An A-DNA triplet code - Thermodynamic rules for predicting A-DNA and B-DNA. Proc Natl Acad Sci USA. 1995;92(14):6464-8.
3. Korkmaz G, Holm M, Wiens T, Sanyal S. Comprehensive analysis of stop codon usage in bacteria and its correlation with release factor abundance. J Biol Chem. 2014;289(44):30334-42.
4. Ho AT, Hurst LD. In eubacteria, unlike eukaryotes, there is no evidence for selection favouring fail-safe 3' additional stop codons. PLoS Genet. 2019;15(9):e1008386.
5. Floydsmith G, Slattery E, Lengyel P. Interferon action - RNA cleavage pattern of a (2'-5')oligoadenylate-dependent endonuclease. Science. 1981;212(4498):1030-2.
6. Wreschner DH, McCauley JW, Skehel JJ, Kerr IM. Interferon action-sequence specificity of the ppp(A2'p)nA-dependent ribonuclease. Nature. 1981;289(5796):414-7.
7. Wahl MC, Will CL, Luhrmann R. The spliceosome: design principles of a dynamic RNP machine. Cell. 2009;136(4):701-18.
8. Wimmer K, Schamschula E, Wernstedt A, Traunfellner P, Amberger A, Zschocke J, et al. AG-exclusion zone revisited: Lessons to learn from 91 intronic NF1 3 ' splice site mutations outside the canonical AG-dinucleotides. Hum Mutat. 2020;41(6):1145-56.
9. Karlin S, Mrazek J. Compositional differences within and between eukaryotic genomes. Proc Natl Acad Sci USA. 1997;94(19):10227-32.
10. Mrazek J, Karls AC. In silico simulations of occurrence of transcription factor binding sites in bacterial genomes. BMC Evol Biol. 2019;19:67.
11. Hahn MW, Stajich JE, Wray GA. The effects of selection against spurious transcription factor binding sites. Mol Biol Evol. 2003;20(6):901-6.
12. Berg OG. Selection intensity for codon bias and the effective population size of Escherichia coli. Genetics. 1996;142(4):1379-82.
